# Prevalence of Prenatal Opioid Exposure in Ontario, Canada, 2014-2019

**DOI:** 10.1001/jamanetworkopen.2020.37388

**Published:** 2021-02-17

**Authors:** Andi Camden, Joel G. Ray, Teresa To, Tara Gomes, Li Bai, Astrid Guttmann

**Affiliations:** 1 Division of Epidemiology, Dalla Lana School of Public Health, University of Toronto, Toronto, Ontario, Canada; 2 Department of Medicine, St Michael’s Hospital, Toronto, Ontario, Canada; 3 Child Health Evaluative Sciences, Research Institute, The Hospital for Sick Children, Toronto, Ontario, Canada; 4 Li Ka Shing Knowledge Institute, St Michael’s Hospital, Toronto, Ontario, Canada; 5 Life Stage Research Program, ICES (formerly the Institute for Clinical Evaluative Sciences), Toronto, Ontario, Canada

## Abstract

This population-based cohort study assesses the prevalence of opioid use in pregnant women in Ontario, Canada, from 2014 through 2019.

## Introduction

Prenatal opioid exposure (POE) is a major public health concern necessitating accurate surveillance data.^1^ Corsi et al^2^ recently completed a large population-based study in Ontario, Canada. Although they estimated that 1.1% of women used an opioid during pregnancy, they did not distinguish between prescribed vs illicit opioids and relied on self-reported registry data.^3^ Other studies of women in Ontario who were prescribed opioids during pregnancy were restricted to women eligible for social assistance or rural geographic areas.^4,5^ To overcome these limitations in the estimation of POE, we used linked health administrative data across Ontario, including all opioid prescriptions, to study the prevalence of POE in pregnant women.

## Methods

This population-based cohort study was completed at ICES (formerly the Institute for Clinical Evaluative Sciences), an independent, nonprofit research institute whose legal status allows collection and analysis of health and demographic data without consent for health system evaluation. Data sets were linked using unique encoded identifiers (eTable in the Supplement). Ethics approval was granted by the research ethics boards of the University of Toronto and Hospital for Sick Children. This study followed the Strengthening the Reporting of Observational Studies in Epidemiology (STROBE) and Reporting of Studies Conducted Using Observational Routinely Collected Data (RECORD) reporting guidelines.

All legal residents in Ontario have universal, publicly funded health care that includes hospital and physician services. We identified all hospitalizations in Ontario from January 1, 2014, through December 31, 2019, for live births or stillbirths among women aged 12 to 50 years who were eligible for provincial health care throughout their pregnancy.

We identified POE using maternal prescription opioid data during pregnancy, categorized as (1) analgesics for pain, (2) opioid for cough, (3) opioid agonist therapy (OAT) for opioid use disorder (methadone and buprenorphine hydrochloride), (4) opioid-related maternal hospital records during pregnancy, (5) newborn records with neonatal abstinence syndrome, and (6) outpatient visits for OAT. We attempted to identify illicit opioid exposure based on (1) a maternal-child record with neonatal abstinence syndrome or (2) maternal opioid-related hospital care during pregnancy, and neither (3) maternal prescription for an opioid nor (4) maternal OAT outpatient visit.

These definitions were used to estimate the prevalence of POE and the type of opioid exposure among all births during the study period. Annual trends were assessed using modified Poisson regression models. Statistical significance was defined as 2-sided *P* < .05. Analyses were performed using SAS, version 9.4 (SAS Institute Inc).

## Results

Hospital births during the study period totaled 701 896, and 37 415 newborns (5.3%) had POE. Mothers were a mean (SD) age of 30.9 (5.3) years at current birth, 74.7% were Canadian-born, and 10.5% had pain-related hospital care 2 years before conception. The annual prevalence of POE decreased from 6.1% in 2014 to 4.5% in 2019 (*P* < .001). The highest rates of POE were among women aged 20 to 24 years (8.8% in 2014 to 7.1% in 2019), those living in rural areas (8.3% in 2014 to 6.6% in 2019), and those residing in neighborhoods in the lowest income quintile (8.3% in 2014 to 6.7% in 2019) (Figure, A). Opioid analgesic use decreased from 4.5% to 3.1% (*P* < .001) and was most pronounced among women for whom an opioid analgesic was prescribed for less than 30 days (Figure, B and C). Overall, dispensed OAT remained stable over time (from 0.7% in 2014 to 0.8% in 2019) (Figure, B). However, methadone use decreased from 0.6% to 0.5% (*P* < .001), whereas buprenorphine use increased from 0.1% to 0.3% (*P* < .001; Figure, D). Rates for illicit opioid use, maternal opioid-related hospital care, and neonatal abstinence syndrome remained stable over time (Figure, B). For example, the rate for neonatal abstinence syndrome was 0.9% in 2014 and 2019 (*P* = .19). The Table details the maternal characteristics by opioid use during pregnancy.

**Figure.**
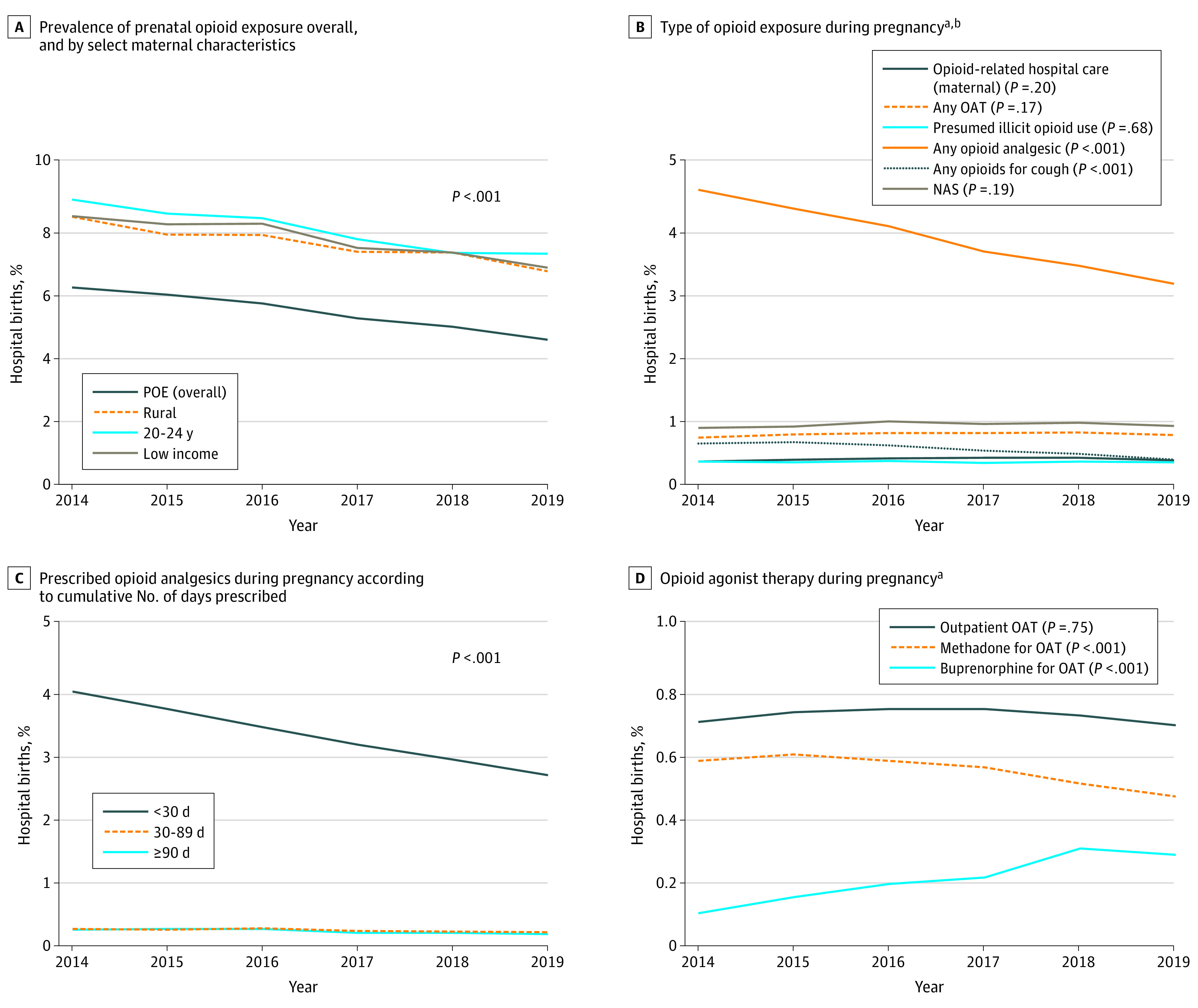
Temporal Trends in Prenatal Opioid Exposure (POE) Among All Hospital Births in Ontario, Canada, 2014-2019 A, Decreases in the overall prevalence of POE over the study period and by women who live in low-income areas, rural areas, and among 20- to 24-year-olds. B, Trends over time by type of opioid exposure, including prescription of opioids, outpatient OAT, and opioid-related hospital care for mothers and newborns. C, Decreases in opioid analgesic use in pregnancy categorized by the number of cumulative days covered in pregnancy by opioid analgesics. D, Opioid agonist therapy use in pregnancy by OAT outpatient visits and prescriptions for methadone or buprenorphine hydrochloride. The number of hospital births was 118 915 in 2014; 118 278 in 2015; 118 242 in 2016; 116 379 in 2017; 115 252 in 2018; and 114 830 in 2019. The number of live hospital births was 118 274 in 2014; 117 634 in 2015; 117 585 in 2016; 115 759 in 2017; 114 656 in 2018; and 114 216 in 2019. *P* values were calculated using modified Poisson regression models. Sources for the data shown are the Narcotics Monitoring System (opioid prescriptions), Discharge Abstract Database (hospitalizations), National Ambulatory Care Reporting System (emergency department visits), Ontario Mental Health Reporting System (mental health hospitalizations), and Ontario Health Insurance Plan (outpatient visits) (eTable in the Supplement). NAS indicates neonatal abstinence syndrome; OAT, opioid agonist therapy. ^a^Type of opioid exposure is not mutually exclusive. ^b^NAS is based on live hospital births.

**Table.** Maternal Characteristics by Opioid Use During Pregnancy Among 701 896 Hospital Births in Ontario, Canada, 2014-2019^a^

Characteristic	Any opioid use, No. (%)^b^				No opioid use, No. (%) (n = 664 481)
	Prescribed opioid		Opioid agonist therapy (n = 5402)^c^	Opioid use presumed illicit (n = 2421)	
	Analgesic (n = 26 752)	For cough (n = 3826)			
Maternal demographic characteristics					
Age, mean (SD), y					
At first delivery	26.6 (6.1)	28.7 (5.4)	22.3 (5.1)	22.3 (5.4)	28.2 (5.5)
At current delivery	30.5 (5.6)	32.0 (5.0)	28.5 (4.8)	27.1 (6.0)	31.0 (5.2)
Area-level lowest income quintile^d^	7151 (26.7)	822 (21.5)	2700 (50.0)	1199 (49.5)	140 241 (21.1)
Rural residence^e^	3234 (12.1)	216 (5.6)	1427 (26.4)	702 (29.0)	68 022 (10.2)
Immigrant or recent OHIP registrant	4339 (16.2)	1562 (40.8)	60 (1.1)	77 (3.2)	171 502 (25.8)
≥3 Previous live births	3053 (11.4)	265 (6.9)	1354 (25.1)	517 (21.4)	44 462 (6.7)
Social risk factors (2 y before conception)					
Infant discharged to social services at birth	294 (1.1)	16 (0.4)	486 (9.0)	415 (17.1)	1758 (0.3)
Violence-related health care use	495 (1.9)	14 (0.4)	324 (6.0)	229 (9.5)	4041 (0.6)
Homelessness^f^	Suppressed	Suppressed	125 (2.3)	93 (3.8)	718 (0.1)
Criminal justice system involvement^f^	317 (1.2)	44 (1.2)	402 (7.4)	168 (6.9)	4758 (0.7)
Substance use–related hospital care (2 y before conception)					
Tobacco-related	772 (2.9)	43 (1.1)	423 (7.8)	161 (6.7)	7070 (1.1)
Alcohol-related	577 (2.2)	27 (0.7)	437 (8.1)	251 (10.4)	4862 (0.8)
Opioid-related	296 (1.1)	8 (0.2)	1120 (20.7)	216 (8.9)	766 (0.1)
Nonopioid or multidrug	451 (1.7)	21 (0.5)	653 (12.1)	326 (13.5)	2994 (0.5)
Medical morbidity (2 y before conception)					
Mental health–related ED visit or hospitalization	1629 (6.1)	104 (2.7)	680 (12.6)	476 (19.7)	15 560 (2.3)
Pain-related hospital care^g^	6857 (25.6)	508 (13.3)	1115 (20.6)	489 (20.2)	68 921 (10.4)
High medical comorbidity	6937 (25.9)	775 (20.3)	1221 (22.6)	478 (19.7)	63 622 (9.6)
Pregnancy-related					
No prenatal care in the first trimester from a physician	936 (3.5)	84 (2.2)	280 (5.2)	696 (28.7)	47 610 (7.2)
No fetal ultrasound by 20 wk	1631 (6.1)	232 (6.1)	1078 (20.0)	654 (27.0)	45 009 (6.8)
Proportion of days in pregnancy covered by prescription opioid use, mean (SD)	0.09 (0.21)	0.05 (0.10)	0.64 (0.36)	NA	NA
Any prescription opioid exposure					
First trimester	12 584 (47.0)	2570 (67.2)	4462 (82.6)	NA	NA
Second trimester	10 898 (40.7)	951 (24.9)	4418 (81.8)	NA	NA
Third trimester^h^	10 577 (39.6)	807 (21.2)	4293 (79.5)	NA	NA

Abbreviations: ED, emergency department; NA, not applicable; OHIP, Ontario Health Insurance Plan.

^a^ Data sources were the Narcotics Monitoring System (opioid prescriptions); Discharge Abstract Database (hospitalizations); National Ambulatory Care Reporting System (emergency department visits); Ontario Mental Health Reporting System (mental health hospitalizations); Ontario Health Insurance Plan (outpatient visits); Immigration, Refugees and Citizenship Canada Permanent Resident (immigration); Registered Persons Database (demographic characteristics); and Postal Code Conversion File (demographic characteristics). Sample sizes smaller than 6 were suppressed.

^b^ Type of opioid exposure was not mutually exclusive.

^c^ Data for use of an opioid agonist therapy were based on any prescriptions for methadone or buprenorphine hydrochloride or for outpatient visits for prescription of an opioid agonist therapy.

^d^ Area-level lowest income quintile was defined as quintile 1.

^e^ Rural residences were defined as communities with fewer than 10 000 inhabitants.

^f^ As noted on health care records; may not be comprehensive.

^g^ Pain conditions include low back pain, migraine, and chronic pain conditions (rheumatoid arthritis, fibromyalgia, joint pain, chronic pancreatitis, peripheral neuropathy, sickle cell disease, and renal calculi).

^h^ Missing data for 3311 preterm births.

## Discussion

The observed rate of POE in our study (5.3% from January 1, 2014, through December 31, 2019) was nearly 5 times higher than that described by Corsi et al^2^ (1.1% from April 1, 2012, through March 31, 2018). Prenatal opioid exposure decreased over time; this decrease was induced primarily by decreases in prescribed opioid analgesics and cough medications. Decreases in the use of opioid analgesics or antitussive agents likely reflect the uptake of more conservative opioid-prescribing guidelines, whereas increases in the use of buprenorphine likely reflect the 2017 Canadian clinical practice guidelines on opioid use disorder management with buprenorphine or methadone.^6^

Our study has limitations. Administrative data were limited to those patients who used the health care system and relied on the accuracy of diagnostic codes, which may have resulted in misclassification bias. Although our findings may underestimate illicit opioid use and include short courses of analgesic opioids, they nevertheless highlight potential underreporting of POE when clinical registries are used that lack granular data on prescribed medications.

## Notes

Supplement. **eTable.** Methods Used in the Current Study to Describe Prenatal Opioid Exposure and Other Variables

## References

[1] Honein MA, Boyle C, Redfield RR Public health surveillance of prenatal opioid exposure in mothers and infants. Pediatrics. 2019;143(3):e20183801. doi:10.1542/peds.2018-3801 30655335 PMC6482836

[2] Corsi DJ, Hsu H, Fell DB, Wen SW, Walker M Association of maternal opioid use in pregnancy with adverse perinatal outcomes in Ontario, Canada, from 2012 to 2018. JAMA Netw Open. 2020;3(7):e208256.doi:10.1001/jamanetworkopen.2020.8256 32725246 PMC12064095

[3] Palamar JJ Commentary on Ondersma et al (2019): will better self-report screening instruments be enough to detect drug use during pregnancy? Addiction. 2019;114(9):1694-1695. doi:10.1111/add.14717 31301078 PMC7046169

[4] Brogly SB, Turner S, Lajkosz K, Infants born to opioid-dependent women in Ontario, 2002–2014. J Obstet Gynaecol Can. 2017;39(3):157-165. doi:10.1016/j.jogc.2016.11.009 28343557

[5] Dooley J, Ryan G, Gerber Finn L, Maternal opioid use disorder and neonatal abstinence syndrome in northwest Ontario: a 7-year retrospective analysis. Can J Rural Med. 2018;23(2):39-44. 29547380

[6] Ordean A, Wong S, Graves L No. 349—substance use in pregnancy. J Obstet Gynaecol Can. 2017;39(10):922-937.e2. doi:10.1016/j.jogc.2017.04.028 28935057

