# Supplementary material for: Prevalence of Prenatal Opioid Exposure in Ontario, Canada, 2014-2019
Source: JAMA Netw Open. 2021 Feb 17;4(2):e2037388. doi: 10.1001/jamanetworkopen.2020.37388 (PMC7890532; doi:10.1001/jamanetworkopen.2020.37388)
Supplement: Supplement. — eTable. Methods Used in the Current Study to Describe Prenatal Opioid Exposure and Other Variables [file jamanetwopen-e2037388-s001.pdf]

Supplementary Online Content

Camden A, Ray JG, To T, Gomes T, Bai L, Guttmann A. Prevalence of prenatal opioid exposure in Ontario, Canada, 2014-2019. *JAMA Netw Open.* 2021;4(2):e2037388. doi:10.1001.jamanetworkopen.2020.37388

eTable. Methods Used in the Current Study to Describe Prenatal Opioid Exposure and Other Variables

This supplementary material has been provided by the authors to give readers additional information about their work.

©2021 Camden A et al. *JAMA Network Open.*

eTable. Methods used in the current study to describe prenatal opioid exposure and other variables

Measure	Definition	Timing	Data source(s)
**Prenatal opioid exposure**			
Prenatal opioid exposure (POE)	Any maternal prescribed opioid during pregnancy OR maternal opioid-related hospital care during pregnancy OR outpatient visits for Opioid Agonist Therapy (OAT) OR neonatal abstinence syndrome (NAS)	Conception to delivery; NAS - Newborn birth episode; re-admission within 14 days of life	NMS, DAD, NACRS, OHMRS, OHIP<br><br>Validation studies have demonstrated these databases are complete and accurate:<br>Canadian Institute for Health Information, CIHI Data Quality Study of the 2006–2007 Discharge Abstract<br>Database (Ottawa, Ont.: CIHI, 2009).<br>Martin L, Hirdes J, Morris J, Montague P, Rabinowitz T, Fries B. Validating the Mental Health Assessment Protocols (MHAPs) in the Resident Assessment<br>Instrument Mental Health (RAI-MH). J Psychiatr Ment Health Nurs 2009; 16:<br>646–53.<br>Schull MJ, Azimaee M, Marra M, Cartagena RG, Vermeulen MJ, Ho M, Guttmann A. ICES: Data, Discovery, Better Health. International Journal of Population Data Science. 2019;4(2).
Any maternal prenatal prescribed opioid	Any prescription dispensed for an opioid analgesic OR methadone OR buprenorphine for OAT OR opioids for cough	Conception to delivery; Trimester	NMS (hyperlink: https://datadictionary.ices.on.ca/Applications/DataDictionary/Library.aspx?Library=NMS)<br><br>Martins, D., Singh, S., Khuu, W., Tadrous, M., Paterson, M., Juurlink, D., Mamdani, M. and Gomes, T. (2018) "Linking the Narcotics Monitoring System Database to Quantify the Contribution of Prescribed and Non-Prescribed Opioids to Opioid Overdoses in Ontario, Canada", International Journal of Population Data Science, 3(4). doi: 10.23889/ijpds.v3i4.644.<br><br>Note: NMS may be missing prescriptions from people without a health card or Indigenous peoples with a status card.

©2021 Camden A et al. *JAMA Network Open.*

Measure	Definition	Timing	Data source(s)
Any maternal prenatal prescription for opioid analgesic for pain	Any prescription for opioid analgesics for pain (morphine, codeine, oxycodone, hydrocodone, hydromorphone, meperidine, tramadol, methadone, buprenorphine, fentanyl, tapentadol)	Conception to delivery; Trimester	NMS
Any maternal prenatal prescription for Opioid Agonist Therapy (OAT)	Any prescription for methadone OR buprenorphine for OAT	Conception to delivery; Trimester	NMS
Any maternal prenatal prescription for methadone	Any prescription for methadone for OAT	Conception to delivery; Trimester	NMS
Any maternal prenatal prescription for buprenorphine	Any prescription for buprenorphine for OAT	Conception to delivery; Trimester	NMS
Any maternal prenatal prescription for opioids for cough	Any prescription for opioids for cough	Conception to delivery; Trimester	NMS
Maternal opioid-related hospital care	ICD-10 F11, T40.0-T40.4, T40.6, DSM-5 304.00, 305.50, Y45.0	2 years prior to conception; Pregnancy (conception to discharge of the delivery record	DAD (hyperlink: https://datadictionary.ices.on.ca/Applications/DataDictionary/Library.aspx?Library=DAD), NACRS (hyperlink: https://datadictionary.ices.on.ca/Applications/DataDictionary/Library.aspx?Library=NACRS), OHMRS (hyperlink: https://datadictionary.ices.on.ca/Applications/DataDictionary/Library.aspx?Library=OMHRS)
Newborn hospital records with NAS	ICD-10 P96.1 – Withdrawal from maternal drugs of addiction OR ICD-10 P04.4 – Infant affected by maternal drugs of addiction	Newborn birth episode; re-admission within 14 days of life	DAD<br>Guttmann A, Blackburn R, Amartey A, Zhou L, Wijlaars L, Saunders N, Harron K, Chiu M, Gilbert R. Long-term mortality in mothers of infants with neonatal abstinence syndrome: A population-based parallel-cohort study in England and Ontario, Canada. PLoS medicine. 2019 Nov 26;16(11):e1002974.

©2021 Camden A et al. *JAMA Network Open.*

Measure	Definition	Timing	Data source(s)
Presumed illicit opioid use	NAS diagnostic codes in newborn hospital record or maternal-opioid related hospital care during pregnancy and no record of prescribed opioids or outpatient OAT visits	Conception to delivery	NMS, DAD, NACRS, OHMRS, OHIP
Outpatient Opioid Agonist Therapy (OAT)	Any of: K682 (Opioid agonist maintenance program monthly management fee – intensive); K683 (Opioid agonist maintenance program monthly management fee – maintenance); K684 (Opioid agonist maintenance program monthly management fee - team maintenance)	Conception to delivery	OHIP
Any OAT	Any of: Outpatient Opioid Agonist Therapy, prescription for methadone, prescription for buprenorphine	Conception to delivery	OHIP, NMS
**Maternal substance use-related health care**			
Any tobacco	ICD-10 F17, T65.2, Z72.0, Z71.6, OHIP DXCODE 305	2 years prior to conception	DAD, NACRS, OHIP (hyperlink: https://datadictionary.ices.on.ca/Applications/DataDictionary/Library.aspx?Library=OHIP)
Any alcohol	OHIP DXCODE: 291, 303, ICD10CA G62.1, G31.2, G72.1, I42.6, K29.2, K85.2, K70, K86.0, E24.4, F10, X45, X65, Y15, T51, R78.0, Y90, Y91, Z72.1, Z71.4	2 years prior to conception	DAD, NACRS, OHIP
Any non-opioid/multi-drug use	ICD-10 F12, T40.7, F13, F14, T40.5, R78.2, F15, T43, F16, T40.8, T40.9, F18, F19, Z72.2, Z71.5, OHIP DXCODE 292, 304	2 years prior to conception	DAD, NACRS, OHIP
**Social factors**			
Homelessness as recorded on health care records	The record meets any following criteria: (1) DAD: ICD-10 Z590 or homeless=Y or postal code=XX; (2) NACRS: ICD-10 Z590 or residence type= Homeless or postal code=XX; (3) OMHRS: usual residence= Homeless (with or without shelter)	2 years prior to conception	DAD, NACRS, OMHRS

©2021 Camden A et al. *JAMA Network Open.*

Measure	Definition	Timing	Data source(s)
Health care received while involved with the criminal justice system as recorded on health care records	The record meets any following criteria:<br>(1) institution in OHIP with institution type =Correctional Centre.<br>(2) institution number in DAD with institution type =Correctional Centre and referred from law enforcement<br>(3)instfnum in NACRS with institution type=Correctional Centre and referred from a Legal service.<br>(4) Last 4 digits of X65 in OMHRS with institution type = Correctional Centre.	2 years prior to conception	OHIP, DAD, NACRS, OMHRS
Violence-related health care use	ICD-10: X85-X99, Y00-Y09, Y871, T740-T749	2 years prior to conception	NACRS, DAD
**Demographics**			
Maternal age at first delivery (years)		Index delivery	MOMBABY (hyperlink: https://datadictionary.ices.on.ca/Applications/DataDictionary/Library.aspx?Library=MOMBABY)
Maternal age at current delivery (years)		Index delivery	MOMBABY
3+ previous livebirths	Number of previous livebirths derived from all mom's records	Index delivery	MOMBABY
Area-level lowest income (Quintile 1)	Groups: Quintile 1 (lowest)-Quintile 5 (highest), missing data (represents suppressed) were re-coded to Quintile.<br>Note: Missing data are suppressed for neighbourhoods with high residential instability, which are most likely to be low income and urban.	Conception	RPDB (hyperlink: https://datadictionary.ices.on.ca/Applications/DataDictionary/Library.aspx?Library=RPDB), PCCF+
Rural residence	<10,000 residents, missing data were re-coded as urban<br>Note: Missing data are suppressed for neighbourhoods with high residential instability, which are most likely to be low income and urban.	Conception	PCCF+

©2021 Camden A et al. *JAMA Network Open.*

Measure	Definition	Timing	Data source(s)
Immigrant to Canada or recent OHIP registrant	Immigrant to Canada or recent OHIP registrant from June 2017-December 2019	Conception	RPDB, IRCC Permanent Resident Database (hyperlink: https://datadictionary.ices.on.ca/Applications/DataDictionary/Library.aspx?Library=CIC) Chiu M, Lebenbaum M, Lam K, et al. Describing the linkages of the immigration, refugees and citizenship Canada permanent resident data and vital statistics death registry to Ontario's administrative health database. BMC medical informatics and decision making. 2016;16(1):135.
**Pre-pregnancy morbidity**			
High medical comorbidities	Johns Hopkins ADG score of 10+	2 years preconception	DAD, NACRS, OHIP
Pain	ICD-10: M54.5 (low back pain) or Abdominal pain (R10), G43, G44 (migraine/headache), rheumatoid arthritis (ICES-derived cohort), M79.7 (fibromyalgia), M25.5, M25.50-M25.59, K07.63, K07.69 (joint pain), K86.0, K86.1 (chronic pancreatitis), E10.41, E11.41, E13.41, E14.41 (peripheral neuropathy), D57.1, D57.2, D57.3, D57.8 (sickle cell disease), N20.0, N20.1, N20.2, N20.9, N21.0, N21.1, N21.8, N21.9, N22.0, N22.8 (renal calculus)	2 years preconception	

Measure	Definition	Timing	Data source(s)
Any mental health hospitalization or emergency department visit	ICD-10 (DAD/NACRS) DX10CODE1= F06-F09, F20-F99 or DX10CODE2-DX10CODE10 = X60-X84, Y10-Y19, Y28 when DX10CODE1 not equal to F06-F99; ICD-9-CM (OMHRS) Any OMHRS (including missing, except for 290.x, 294.x in primary diagnosis). If primary dx missing and provisional=17, exclude; OMHRS prior to 2016/17 Any OMHRS (including missing, except for 290.x, 294.x in primary diagnosis). If primary dx missing and provisional=2, exclude Self-harm, anxiety, mood disorder, schizophrenia	2 years preconception	DAD, NACRS, OMHRS
**Pregnancy-related variables**			
Fetal ultrasound by 20 weeks	OHIP FEECODE: J157-J160, J457-460	Conception to delivery	OHIP
Prenatal care visit in the first trimester	Fee Code in (A, K, P003, P004, P005) and billed by an MD with Speciality Code 00 (General Practice), or Code 20 (Obstetrics and Gynaecology)	Conception to delivery	OHIP
Proportion of days in pregnancy covered by prescription opioid use	Number of days in pregnancy covered by prescription opioid use / total number of days in pregnancy	Conception to delivery	NMS

Abbreviations: IRCC=Immigration, Refugees and Citizenship Canada Permanent Resident; DAD=Discharge Abstract Database; NACRS=National Ambulatory Care Reporting System; OHIP=Ontario Health Insurance Plan; OMHRS=Ontario Mental Health Reporting System; NMS=Narcotics Monitoring System; RPDB=Registered Persons Database; PCCF+=Postal Code Conversion File.

©2021 Camden A et al. *JAMA Network Open.*
